# Evidence for a Higher Number of Species of *Odontotermes* (Isoptera) than Currently Known from Peninsular Malaysia from Mitochondrial DNA Phylogenies

**DOI:** 10.1371/journal.pone.0020992

**Published:** 2011-06-08

**Authors:** Shawn Cheng, Laurence G. Kirton, Jothi M. Panandam, Siti S. Siraj, Kevin Kit-Siong Ng, Soon-Guan Tan

**Affiliations:** 1 Tropical Forest Biodiversity Centre, Forest Research Institute Malaysia, Kepong, Selangor, Malaysia; 2 Faculty of Agriculture, Universiti Putra Malaysia, Serdang, Selangor, Malaysia; 3 Faculty of Biotechnology and Biomolecular Sciences, Universiti Putra Malaysia, Serdang, Selangor, Malaysia; Natural History Museum of Denmark, Denmark

## Abstract

Termites of the genus *Odontotermes* are important decomposers in the Old World tropics and are sometimes important pests of crops, timber and trees. The species within the genus often have overlapping size ranges and are difficult to differentiate based on morphology. As a result, the taxonomy of *Odontotermes* in Peninsular Malaysia has not been adequately worked out. In this study, we examined the phylogeny of 40 samples of *Odontotermes* from Peninsular Malaysia using two mitochondrial DNA regions, that is, the 16S ribosomal RNA and cytochrome oxidase subunit I genes, to aid in elucidating the number of species in the peninsula. Phylogenies were reconstructed from the individual gene and combined gene data sets using parsimony and likelihood criteria. The phylogenies supported the presence of up to eleven species in Peninsular Malaysia, which were identified as *O. escherichi*, *O. hainanensis*, *O*. *javanicus*, *O*. *longignathus*, *O. malaccensis*, *O. oblongatus*, *O*. *paraoblongatus*, *O*. *sarawakensis*, and three possibly new species. Additionally, some of our taxa are thought to comprise a complex of two or more species. The number of species found in this study using DNA methods was more than the initial nine species thought to occur in Peninsular Malaysia. The support values for the clades and morphology of the soldiers provided further evidence for the existence of eleven or more species. Higher resolution genetic markers such as microsatellites would be required to confirm the presence of cryptic species in some taxa.

## Introduction

Termites of the genus *Odontotermes* belong to the Macrotermitinae, the only subfamily within the Termitidae that cultivates fungus gardens of the basiodiomycete *Termitomyces* to degrade plant material for food. The fungus is cultivated by the termite on combs of faecal material [1]. Apart from the Macrotermitinae, only two other insect lineages, ants and beetles, have independently evolved the ability to cultivate fungi for food. The centre of diversity of the Macrotermitinae is in the forests of tropical west and central Africa [2]. The *Odontotermes* together with *Macrotermes* and *Microtermes* are widely distributed throughout the Afro- and Indotropics. The *Odontotermes* specifically feature as important decomposers in the Old World tropics [3] and as pests of agriculture and forestry in Africa [4]. In Southeast Asia, they have occasionally been found as pests of seedlings and saplings in enrichment plantings of timber tree species in logged-over forests in Malaysia where they damage the bark and roots of young trees, sometimes causing their death [5]. In-depth research on the taxonomy and phylogenetics of the *Odontotermes* has been conducted on the African taxa. Behaviour, nest architecture, soldier morphology and molecular phylogenetics have been used to delineate some of the African species [6], [7], [8]. Mitochondrial DNA (mtDNA) studies have shown that some African populations are morphologically similar but have divergent 16S ribosomal RNA (16S) sequences, while others are morphologically different but have almost identical gene sequences [7], [8].

In Southeast Asia, little work has been done on the taxonomy of the *Odontotermes*. Morphological separation of *Odontotermes* species has largely been based on differences in the size and shape of the head capsule and the position of the marginal tooth on the left mandible of the soldier caste [9], [10]. They do not build mounds in Malaysia and are mainly collected from rotting wood material. Often, workers but little or no soldiers are collected, and alates are rarely ever encountered with them. These characteristics make it difficult to undertake comprehensive taxonomic studies based on their behaviour and morphology. A brief account of the species of *Odontotermes* in Peninsular Malaysia is given in a monograph on termites of this region [11]. No measurements are provided but small figures of the head of soldiers drawn to scale are given for each morphospecies recognised, which are listed against their possible taxa. Identification of the species within the genus was said to be difficult because many species had been described based on small differences that may be accounted for by intra-specific variation. A more detailed account of the *Odontotermes* of Thailand is given by Ahmad [12], however, the reliability of the identifications may still be hampered by a limited understanding of the degree of variation within each species, as pointed out by Tho [11]. In this study, we sequenced fragments of the mitochondrial genes, specifically, the 16S rRNA (16S) and cytochrome oxidase subunit I (cyt1), to elucidate species boundaries within the *Odontotermes* in Peninsular Malaysia and identified named taxa with which they may be associated. In addition, the evolutionary history of the Peninsular Malaysian *Odontotermes* was reconstructed.

## Materials and Methods

**Figure 1:**
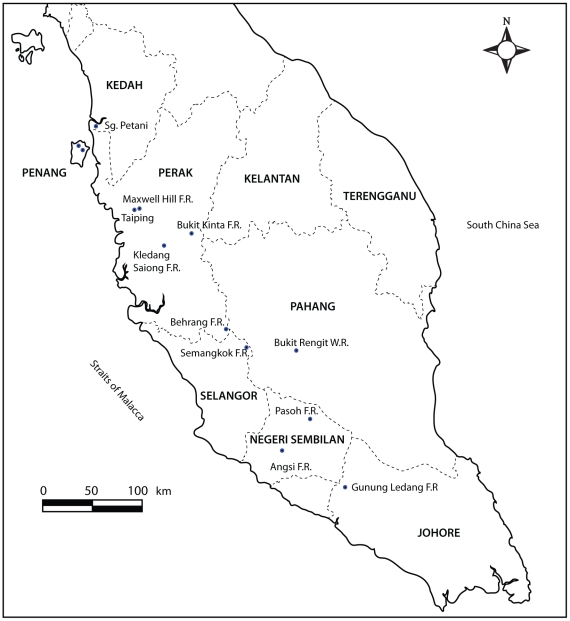
Site locations in Peninsular Malaysia where sampling was conducted (marked with blue dots).

*Odontotermes* were sampled from nine dipterocarp rainforests in the northern, central and southern sections of Peninsular Malaysia (Figure 1). Most of the sites were in forest or wildlife reserves, and their names and GPS coordinates are as follows: Ulu Kinta (N4, 39.036; E101, 14.963); Kledang Saiong (N4, 32.106, E101, 0.158); Bukit Larut (N4 52.101 E100 46.675); Semangkok (N3, 37.358; E101, 44.160); Behrang (N3, 47.422; E101, 33.204); Bukit Rengit (N3, 35.667; E102, 10.785); Pasoh (N2 58.938 E102 18.431); Angsi (N2 42.165 E102 03.389); and Gunung Ledang (N2 21.443 E102 37.279). The samples obtained from these sites were for a systematic ecological study in which all *Odontotermes* were sampled from ten randomly placed 10×10 m plots along a 1-km transect in which live trees and dead plant materials such as logs, stumps and fallen branches, were examined. We also collected *Odontotermes* from other habitats, such as urban parks and beach forests to include as many species as we could in our data set. Wherever possible, soldiers were collected together with workers. Samples were preserved in 75% and 100% undenatured ethanol for morphological and molecular analysis, respectively. They were later examined under a binocular microscope, measured and matched as far as possible to described species using the keys or descriptions of Ahmad [12], Chhotani [13], Holmgren [9] and Tho [11]. All samples are deposited in the Entomological Reference Collection of the Forest Research Institute Malaysia.

The DNA of 40 *Odontotermes* samples representing a range of morphologies were successfully extracted with Wizard® Genomic DNA Purification Kit (Promega, Corp.), from single termite soldiers where possible and occasionally from single workers. The 16S gene was amplified with the primer pair 16Sar and 16Sb developed by Liu & Beckenbach [14]. An additional primer set we developed, Odo16F and Odo16R, amplified the same fragment in several other samples that could not be amplified with the prior mentioned primer set. The cyt1 gene was amplified as two fragments that were later combined to obtain a single length gene using the primer sets developed by Aanen *et al.* [15]. We also sequenced the cytochrome oxidase subunit II (cyt2) gene using the primer pairs described by Liu & Beckenbach [14] and Simon *et al*. [16]. However, the numerous stop codons in the sequences of almost all the samples indicated the presence of pseudogenes in their genomic DNA, thus we excluded it from further analyses. Details of the primers used in the study are provided in the online supporting information in Table S1.

Each gene region was amplified in a polymerase chain reaction (PCR) cocktail containing 2.0 μl DNA (5 ng/µl), 2.0 µl dNTPs (2.0 mM), 2.0 µl 10×PCR buffer, 1.2 µl MgCl_2_ (25 mM), 0.6 µl of each primer (10.0 µM), 11.4 µl distilled water and 0.2 µl GoTaq™ DNA polymerase (Promega, Corp.). PCR was performed in a GeneAmp® 9700 thermal cycler (Applied Biosystems) using the following thermal cycling parameters: initial denaturation at 94°C for three minutes, followed by 36 cycles of denaturation at 94°C for 30 s, annealing at temperatures specific for each primer pair for 30 s (see Appendix 2), extension at 72°C for 1 min, and a final extension at 72°C for 10 min. The PCR product was electrophoresed on 2% agarose gel and visualised under ultraviolet light after ethidium bromide staining, to confirm the presence of bands. The PCR product was then purified using QIAquick® PCR Purification kit, spin column protocol (Qiagen Inc.) and eluted by adding 30 µl of elution buffer EB (10 mM Tris-HCL, pH 8.5) at the final step. The purified DNA was analysed on a 2.0 % agarose gel, and 2.0 µl of the purified product was then used in Big-Dye® Terminator ver 3.1 cycle sequencing reactions on 3130*xl* Genetic Analyzer (Applied Biosystems). Sequencing was performed in the forward and reverse directions.

Sequences were assembled with Sequencher ver. 4.9 (Gene Codes Corp., Ann Arbor, MI), and the coding gene fragments were examined for stop codons, before we obtained consensus sequences for each of the samples. The sequences were then aligned with the dipteran *Drosophila yakuba* (Diptera) mitochondrial genome sequence (accession number X03240.1 from GenBank) to determine the start and end positions of the gene sequences. We also identified the number of haplotype sequences for the 16S and cyt1 genes with the program Collapse ver 1.2 [17]. Gene sequences were then aligned with the ClustalW algorithm in the software BioEdit ver. 7.0.9 [18], and alignments were later checked by eye for accuracy. In reconstructing the phylogeny of the Peninsular Malaysian *Odontotermes*, we included related sequences of the 16S and cyt1 genes from the GenBank DNA sequence database, namely of *O. billitoni* Holmgren, *O. formosanus* (Shiraki), *O. hainanensis* (Light), *O. javanicus* Holmgren, *O. oblongatus* Holmgren, *O. sarawakensis* Holmgren, and *O. minutus* Amir, a majority of which came from the Indotropical region. This was to examine the relatedness of our samples to species found in neighbouring regions. We used a few outgroup species in the individual gene analyses and rooted the trees using a species of *Cubitermes* (Termitidae), a non-fungus growing termite genus. Table S2 in the online supporting information lists the information on the samples used in the DNA study, including collection locality, 16S and cyt1 GenBank accession numbers and species identities.

As species on GenBank rarely had sequences for both gene fragments, we conducted separate analyses for each gene and also concatenated the sequences of both genes for our samples and an outgroup (*Macrotermes subhyalinus* (Rambur) with 16S and cyt1 sequences from a single termite isolate) to reconstruct the combined gene tree of the Peninsular Malaysian *Odontotermes*. Phylogenies were inferred using maximum parsimony and likelihood methods. Parsimony estimates of the phylogeny for the data sets were obtained using PAUP*4.0b [19] while likelihood estimates of the phylogenies were obtained using the program/algorithm RAxML ver 7.0 [20] at the Cyberinfrastructure for Phylogenetic Research (CIPRES) Portal ver. 2.0 (http://www.phylo.org/).

## Results

### Phylogenetic Analyses of the *Odontotermes*

**Figure 2:**
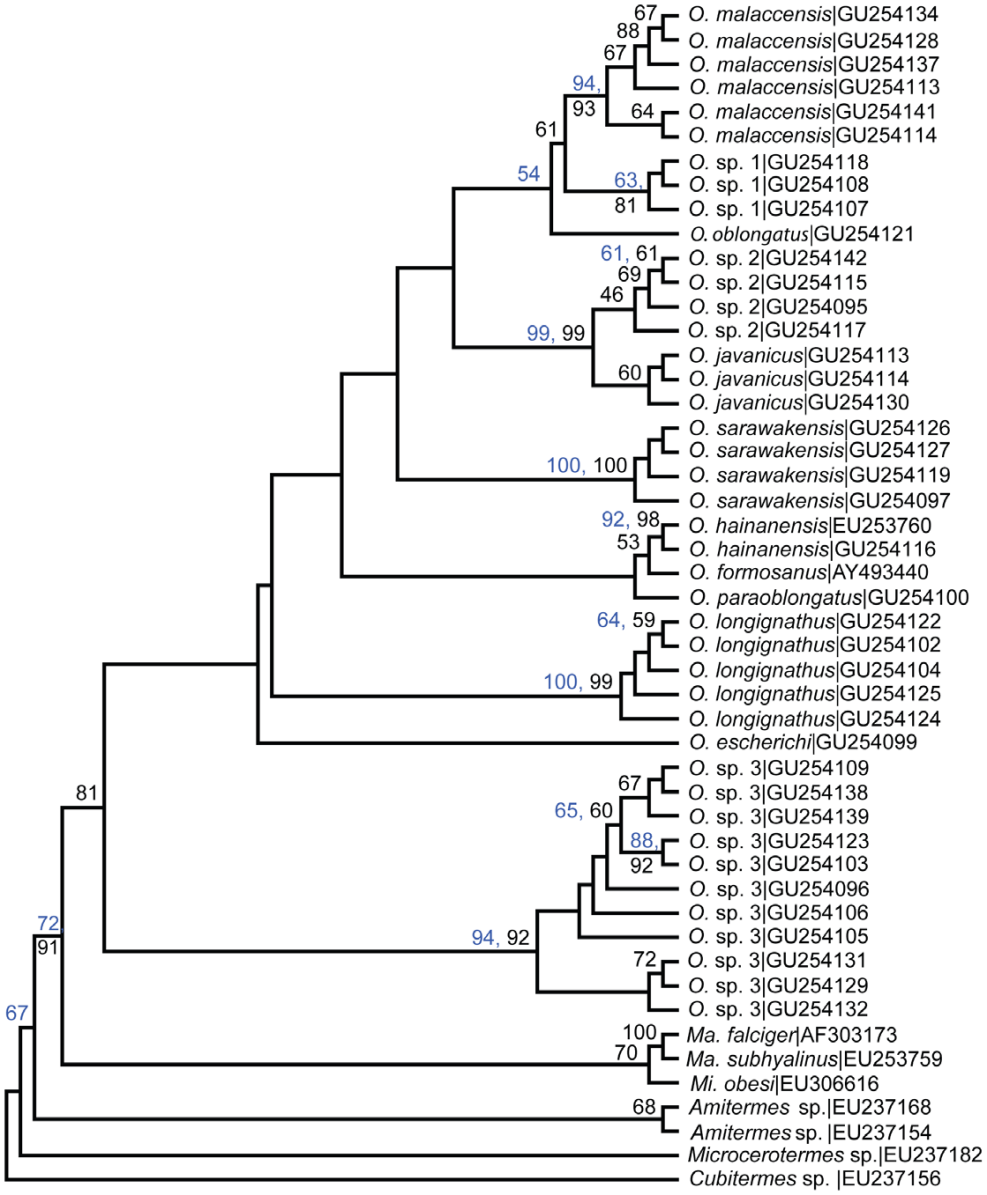
16S maximum likelihood tree of the *Odontotermes* sequences used in the study. GenBank accession numbers follow species names. Bootstrap support values for parsimony and likelihood analyses are in blue and black fonts respectively. Only bootstrap values above 50% are shown for the parsimony analysis.

**Figure 3 pone-0020992-g003:**
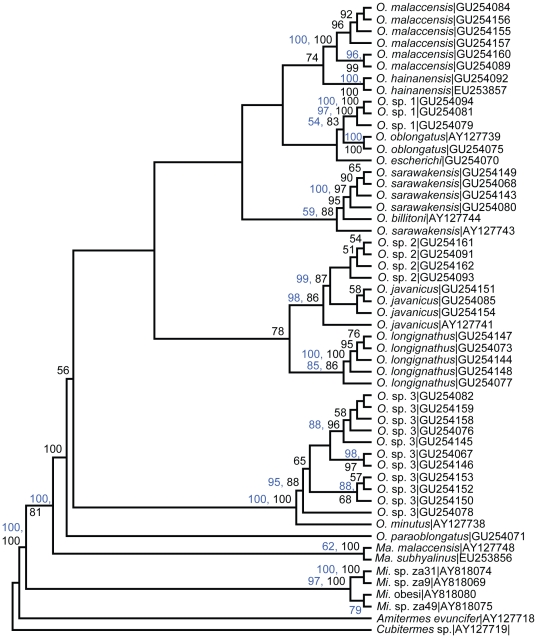
Cyt1 maximum likelihood tree of *Odontotermes* sequences used in the study. Conventions as in **Fig 2**.

The 16S and cyt1 sequences of our specimens returned a closest match to species within the genus *Odontotermes* when queried against nucleotide sequences in the GenBank nucleotide collection database using the BLASTn program. This confirmed the identity of the DNA material extracted. The 16S gene, which is a non-coding and less conserved gene, and the more conserved cyt1 gene, both recovered 22 haplotype sequences (Table S2, online supporting information). The parsimony and likelihood analyses for the separate gene analyses of the 16S and cyt1 recovered the same clades of species although the relationships between them were slightly different. The likelihood trees of the individual gene analyses, drawn with equal branch lengths, are shown in Figures 2 and 3. Parsimony trees are included in supplementary data in Figures S1 & S2.

#### 16S phylogeny

The 16S phylogenetic tree in Figure 2 shows that the *Odontotermes* form an in-group with twelve clades or species that included non-Peninsular Malaysian samples. Although most of the taxa could be identified from their soldier morphologies, morphospecies numbers (sp. 1, etc.) are provided for three species that could not be identified. Clustering of one our samples (GenBank sequence GU254116) with an *O. hainanensis* sequence from China (EU253760) helped us recognise the presence of this species in our samples; its identification was later verified against descriptions and size measurements of the species. This species has not been previously recorded from Peninsular Malaysia. The taxon designated as *O.* sp. 3 was initially identified as *O. formosanus* as it matched the description and size ranges given by Holmgren [9] and Ahmad [12]. However, our taxon did not cluster with the GenBank sample of *O. formosanus* but appeared on a different clade of the tree (Figure 2).

#### Cyt1 phylogeny

In the cyt1 tree, up to thirteen taxa formed the ingroup which comprised the *Odontotermes* (Figure 3). *O. hainanensis* was recovered again, with the Peninsular Malaysian sequence forming a clade with a sequence of this species from China (EU253857). Peninsular Malaysian samples that were identified as *O. sarawakensis* formed a clade with the Bornean samples of *O. sarawakensis* (AY127743) and *O. billitoni* (AY127744) from GenBank. It is uncertain whether these samples all belonged to the same species. In the cyt1 tree, *O.* sp. 3, clustered with an *O. minutus* sample from Borneo.

#### 16S+Cyt1 phylogeny

**Figure 4:**
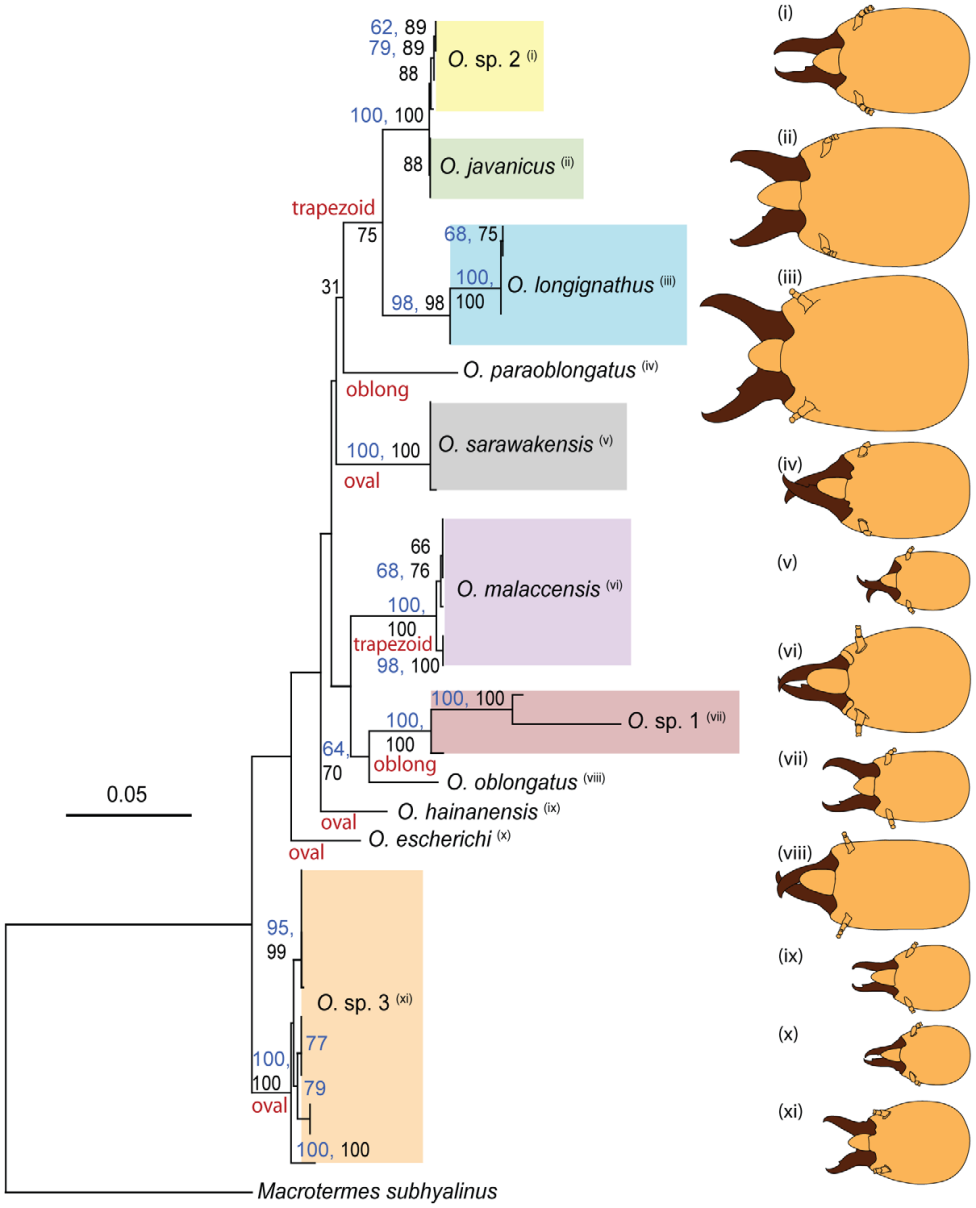
Maximum likelihood tree of the *Odontotermes* constructed from 16S and cox1 genes. Line drawings of soldiers of each species are shown on the right. Vertical bars of different colours represent species clades. Head shape types are given on branches.

The combined gene tree is shown in Figure 4 against illustrations of the soldiers. The combined gene tree supported the existence of at least eleven species of *Odontotermes*. Two clades, namely *O. longignathus* Holmgren and *O.* sp. 1 contained two or more highly divergent morphotypes that could well be different species. *Odontotermes* sp. 2 was closely related to *O. javanicus* in the combined gene tree as well as in the 16S and cyt1 trees, but although they appeared very close genetically, they formed two separate clades (Figures 2 to [Fig pone-0020992-g003] 4). The species are morphologically distinguishable on the basis of a large size difference (Figure 4). *Odontotermes* sp. 2 bears a resemblance to *O. feae* Wasmann in Ahmad [12], but the soldiers are outside the size range given by Ahmad. It overlaps in size with specimens we identified as *O. malaccensis* Holmgren but has a slightly different head shape and clustered on a different part of each tree (Figures 2 to [Fig pone-0020992-g003] 4). *Odontotermes* sp. 3 had a basal position in the combined gene tree, a position that was also recovered in the individual gene trees (Figures 2 to [Fig pone-0020992-g003] 4). *O. longignathus*, *O. javanicus* and *O.* sp. 2 were placed apically in the combined gene tree. The combined gene tree showed that the basal taxa tended to have smaller-sized soldiers while the apically-positioned ones had larger-sized soldiers as indicated by the size of the soldier head capsules shown in Figure 4.

### Degree of Sequence Divergence between Species of *Odontotermes*

The degree of sequence divergence between Peninsular Malaysian species of *Odontotermes* calculated from the 16S and cyt1 genes are shown in Tables S3 and S4 (online supporting information). In the 16S gene, the differences ranged from as few as three to as many as twenty-nine nucleotides whereas for the cyt1 gene it ranged between one and seventy nucleotides. This was a percentage divergence of between 0.63 to 6.57% for the 16S and 0.10 to 7.86% for the cyt1. Percentage divergence between the genetically related *O.* sp. 2 and *O. javanicus* was particularly low at 0.63% for the 16S and 0.10% for the cyt1 but they are morphologically distinguishable by size. Percentage divergence between the 16S sequences of *O.* sp. 1 and *O. malaccensis* was also relatively low compared to other species pairs, at 1.44% (Table S3). However, for the cyt1 the figure was 6.39%, indicating that the information content in different gene sequences may differ between taxa (Table S4). These two species differed in morphology but shared a sister group relationship in all the trees (Figures 2 to [Fig pone-0020992-g003] 4). Besides the pairs of *O.* sp. 2 and *O. javanicus*, and *O.* sp. 1 and *O. malaccensis*, the rest of the species pairs showed higher pairwise divergence levels of between 2.66 to 6.57% for the 16S, and 5.20 to 7.86% for the cyt1.

### Morphological Patterns in the *Odontotermes*

Three general head shapes of the soldiers were recognised in dorsal profile (Figure 4). Species such as *O. longignathus* had trapezoid heads, wider posteriorly than anteriorly and narrowing gradually. Species such as *O. sarawakensis* had oval heads, rounded at the base and narrow towards the base of the mandibles. In species like *O. paraoblongatus* Ahmad, the head was oblong, that is, nearly rectangularor parallel-sided but rounding slightly towards the base of the mandibles and the back of the head. In *O. oblongatus*, this characteristic is marked while in others it may vary somewhat, bordering on the other shapes. *O. longignathus*, *O. javanicus* and *O*. sp. 2 shared a similar descent (Figure 4) as well as a common character not found in the other species. Their apical tooth on the left mandible occurs near the base of the mandible, whereas in the other species it was either at the mid-point or at the anterior third. The combined gene tree showed that the basal taxa tended to have smaller-sized soldiers while the apically-positioned ones had larger-sized soldiers, as can be seen from the size of the soldier head capsules in Figure 4.

## Discussion

The clades in the trees in Figures 2 to [Fig pone-0020992-g003] 4 sometimes contained a single termite species, and in other cases two or more species. To a large extent, species recognised using the gene trees were similar to those recognised by morphology. Identifications using soldier morphologies yielded nine species, while the existence of at least eleven species was inferred from the phylogenetic trees. However, before we reconstructed the phylogenetic trees, we suspected that there were samples we had initially identified as *O. sarawakensis* that were slightly different in morphology. After reconstructing their phylogenies, it became apparent that the samples were genetically distinct. The first sample was identified as *O. hainanensis* after it was found clustering with a GenBank sequence of *O. hainanensis* in the 16S and cyt1 trees while the other was later identified as *O. escherichi* (Holmgren) when it consistently resolved separately from the rest of the samples (Figures 2 & 3).

The 16S and cyt1 trees strongly supported the existence of the species we identified as *O. malaccensis*, *O. javanicus*, *O. sarawakensis* and *O. longignathus* (Figures 2 & 3). *Odontotermes* sp. 3 was consistently recovered at a basal position in the 16S, cyt1 and combined gene tree (Figures 2 to [Fig pone-0020992-g003] 4). Although initially identified as *O. formosanus*, it failed to cluster with a sequence of this species from China (Figures 2 & 3). In the cyt1 tree it clustered with *O. minutus* from Kalimantan (Figure 3). Until specimens of *O. minutus* are examined, we cannot arrive at a firm conclusion as to their conspecificity. Chhotani [13] has clarified that an earlier record of *O. formosanus* from Arunachal Pradesh (India) by Roonwal and Chhotani [21] was incorrect and excluded the species from a review of the termite fauna of India. The taxon from Peninsular Malaysia is also unlikely to be *O. formosanus*. The morphologies of the two species are, however, very similar and an examination of taxa from a wider geographic range is needed before it can be confirmed whether it is a new species. In the morphotypes designated *O.* sp. 1 and *O*. sp. 2, there were no species that matched the descriptions of these forms in the various published taxonomic accounts of termites from Malaysia and nearby regions such as Thailand or India and they also did not cluster with any identified sequences from GenBank. The usefulness of DNA barcoding is clearly seen here. Phylogenies can help indicate the identity of unknown species and assist in forming hypotheses on the taxonomic status of a species which can then be confirmed by further morphological analysis and examination of the types.

The combined gene tree showed that the *O. longignathus* and *O*. sp. 1 clades contained highly divergent lineages that possibly constituted cryptic species (Figure 4). Soldier specimens of *O.* sp. 1 do not come close to the measurements of any of the species described by Ahmad [12] or illustrated in Tho [11], and there was variation in the shape of the head capsules of soldier specimens from different samples. Our limited specimens of *O. longignathus* do not allow us to infer much from the morphology of the two morphotypes in this clade. A very similar species that has been described is *O. praevelens* (John) but its status as a species distinct from *O. longignathus* is uncertain [11]. This can only be confirmed after type materials are examined and when more samples and DNA sequences of taxa within the *O. longignathus* and *O*. sp. 1 clades are obtained. Some of the taxa such as *O. escherichi*, *O. paraoblongatus, O. hainanensis* and *O. oblongatus* were rare species and this was the reason why singletons were used in the phylogenetic analyses. Nevertheless, the gene trees recovered their positions as distinct species within the *Odontotermes*.

Although the head shapes of the soldiers are useful for differentiating between *Odontotermes* species, they did not correspond with natural groupings. However, the location of the apical tooth on the left mandible near the base of the mandible in *O. longignathus*, *O. javanicus* and *O*. sp. 2 reinforced the common descent of these taxa shown by the phylogenetic tree. The tree also shows that the apically-placed or more evolved species generally had larger-sized soldiers (Figure 4).

### Conclusion

The phylogenies indicate the presence of at least eleven species of *Odontotermes* in Peninsular Malaysia, of which eight were identified as *O. escherichi*, *O. hainanensis*, *O*. *javanicus*, *O*. *longignathus*, *O. malaccensis*, *O. oblongatus*, *O*. *paraoblongatus* and *O*. *sarawakensis* and three were unidentifiable morphospecies. Two of the species, *O. escherichi* and *O. hainanensis*, were new records for Peninsular Malaysia. The three unidentified morphospecies are possibly undescribed. In one species (*O*. sp. 1) there were indications that it is a complex of two or more species based on their cyt1 sequence divergence and slight morphological differences in the morphology of the soldiers. There were also indications of a cryptic species in *O. longignathus*. We have recently developed microsatellite markers for the *Odontotermes* to test for the existence of cryptic species in these groups (unpublished data). Further analysis of the morphology of these putative species is also required prior to a formal taxonomic description.

## Supporting Information

Figure S1 16S consensus tree constructed using the parsimony criterion. The tree was a summary of 32,700 trees with an equal length of 615 steps. The 16S data set comprised a 50-taxa data matrix with 570 characters of which 341 were parsimony informative. The starting tree was obtained via stepwise addition and taxa were added according to the closest addition sequence. Bootstrap values on tree nodes were obtained after 1,000 bootstrap replicates.
(TIF)

Figure S2 Cyt1 consensus tree constructed using the parsimony criterion. The tree was a summary of 100 trees with an equal length of 1,025 steps. The cyt1 data set comprised a 54-taxa data matrix with 1,191 characters of which 284 were parsimony informative. Conventions as in Figure S1.
(TIF)

Table S1 PCR primer sets and annealing temperatures Ta used for the amplification of mtDNA genes.
(DOCX)

Table S2 List of specimens used in the analysis and their GenBank accession numbers.
(DOCX)

Table S3 Nucleotide differences between 16S sequences of *Odontotermes* species.
(DOCX)

Table S4 Nucleotide differences between cyt1 sequences of *Odontotermes* species.
(DOCX)
