# Supplementary figures and images for: Evidence for a Higher Number of Species of Odontotermes (Isoptera) than Currently Known from Peninsular Malaysia from Mitochondrial DNA Phylogenies
Source: PLoS One. 2011 Jun 8;6(6):e20992. doi: 10.1371/journal.pone.0020992 (PMC3110805; doi:10.1371/journal.pone.0020992)

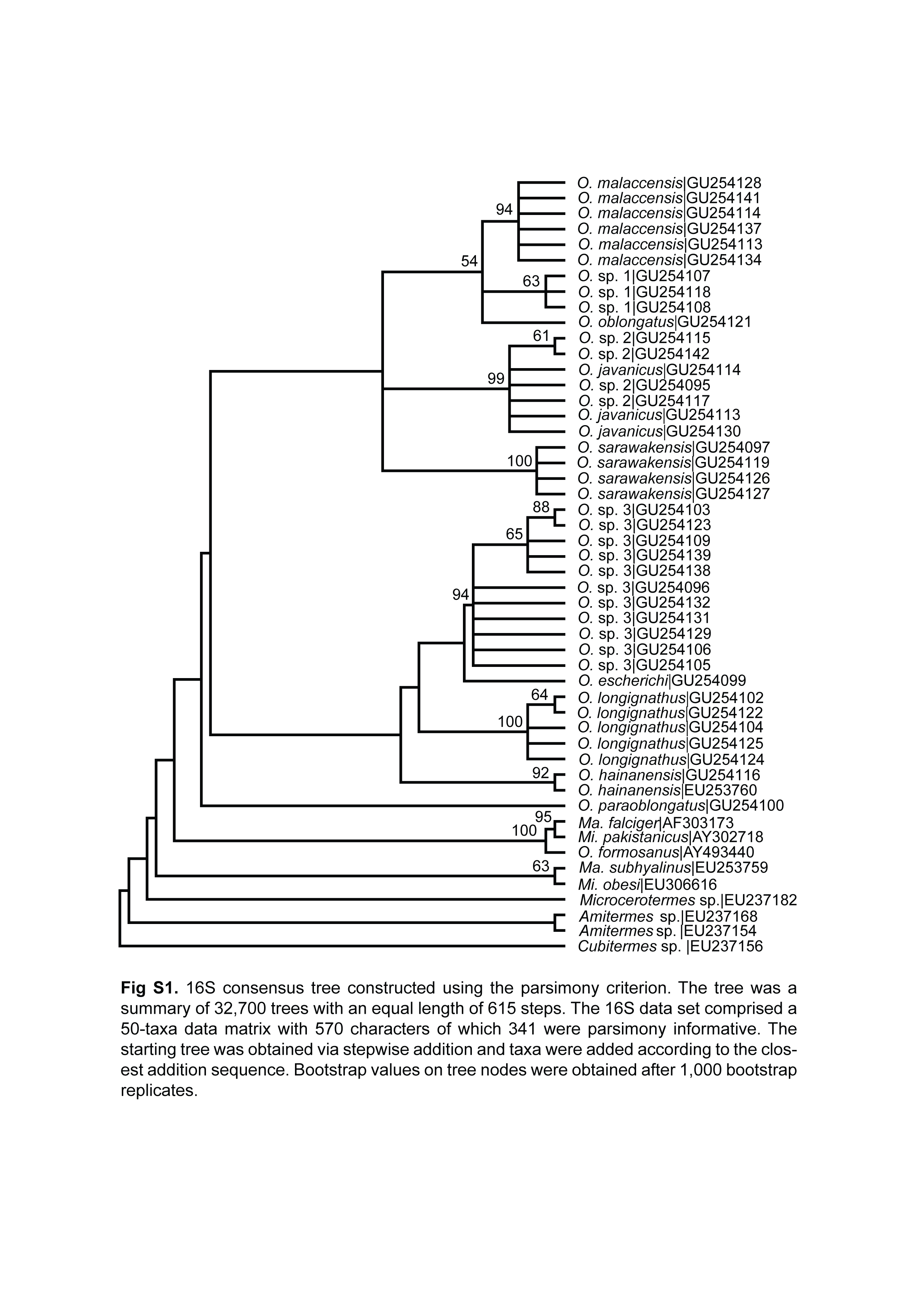

Supplement: Figure S1 — 16S consensus tree constructed using the parsimony criterion. The tree was a summary of 32,700 trees with an equal length of 615 steps. The 16S data set comprised a 50-taxa data matrix with 570 characters of which 341 were parsimony informative. The starting tree was obtained via stepwise addition and taxa were added according to the closest addition sequence. Bootstrap values on tree nodes were obtained after 1,000 bootstrap replicates. (TIF) [file pone.0020992.s001.tif]

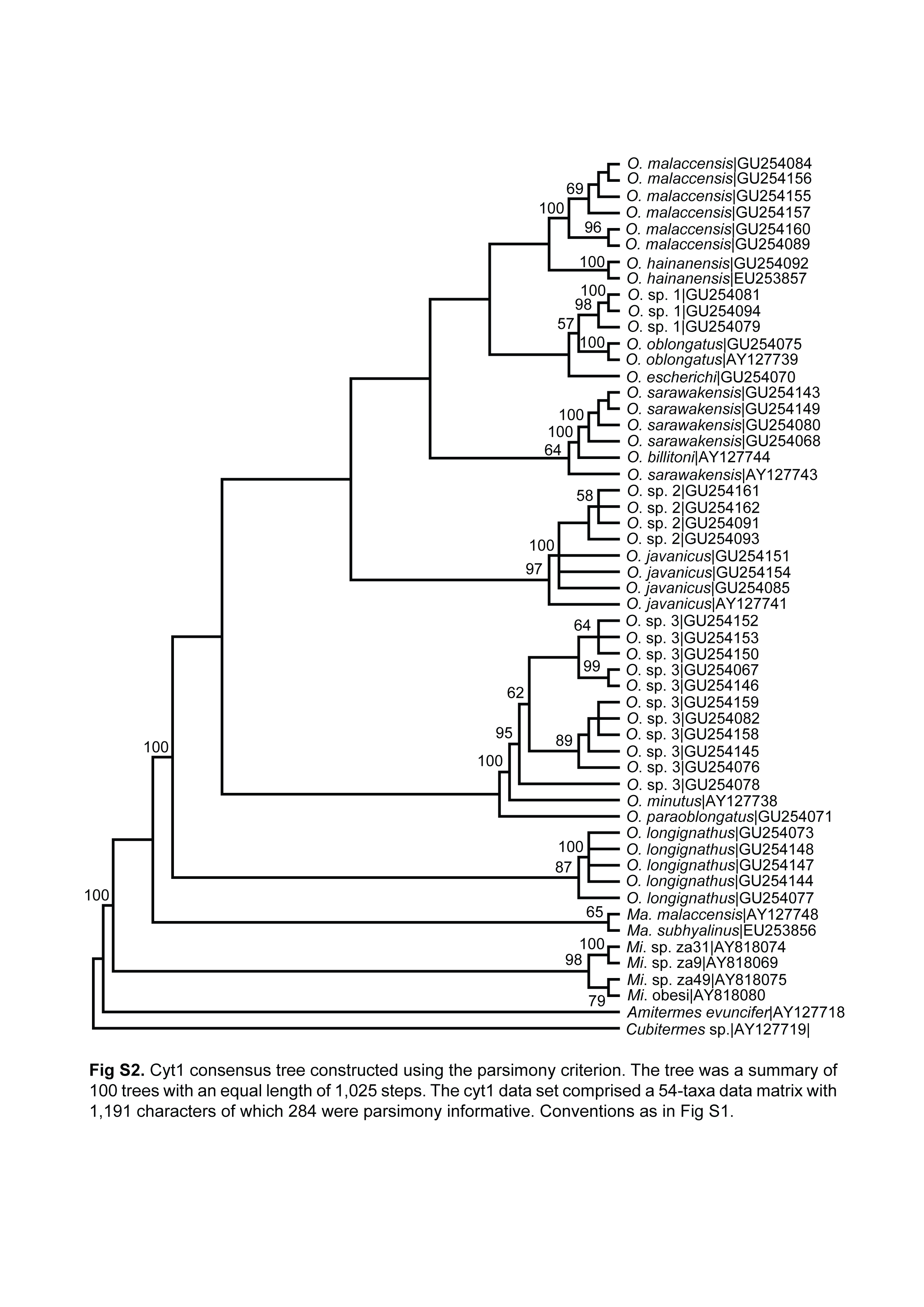

Supplement: Figure S2 — Cyt1 consensus tree constructed using the parsimony criterion. The tree was a summary of 100 trees with an equal length of 1,025 steps. The cyt1 data set comprised a 54-taxa data matrix with 1,191 characters of which 284 were parsimony informative. Conventions as in Figure S1. (TIF) [file pone.0020992.s002.tif]
