# Supplementary material for: Evidence for a Higher Number of Species of Odontotermes (Isoptera) than Currently Known from Peninsular Malaysia from Mitochondrial DNA Phylogenies
Source: PLoS One. 2011 Jun 8;6(6):e20992. doi: 10.1371/journal.pone.0020992 (PMC3110805; doi:10.1371/journal.pone.0020992)
Supplement: Table S1 — PCR primer sets and annealing temperatures Ta used for the amplification of mtDNA genes. (DOCX) [file pone.0020992.s003.docx]

**Table S1.** PCR primer sets and annealing temperatures T_a_ used for the amplification of mtDNA genes.

| Gene | Primer | T_a_ (°C) |
| --- | --- | --- |
| 16S (primer set A)^a^ | 16 Sar (5’-CGCCTGTTTATCAAAAACAT-3’) &  16 Sb (5’-CCGGTCTGAACTCAGATCACGT-3’) | 58.0 |
| 16S (primer set B) | Odo16F (5'-ACGTAAGATTTTAAAGGTCGAA-3') &  Odo16R (5'-ACATCTTTTCTTGTTAGTCTCTGAA-3'). | 58.0 |
| cyt1 (fragment 1, primer set C)^b^ | B11834 (5’-TCAACAAATCATAAAGATATTGG-3’) &  TH2472 (5’-AATAGGTGTTGGTATAGGAT-3’); | 55.3 |
| cyt1 (fragment 2, primer set C)^b^ | TL2350 (5’-CCMCTRTTYGTATGATCAGT-3’) &  TH2877 (5’-GTRTCRTGTARTACRATGTC-3’). | 57.0 |
| cyt1 (fragment 1, primer set D)^b^ | TH2397 (5’-TTAGTAGTATTGTGATTGCTCC-3’) &  TL1862 (5’-TACTTCGTATTCGGAGCTTGA-3’); | 57.0 |
| cyt1 (fragment 2, primer set D)^b^ | TL2341 (5’-CGAACGAATCCCACTATTTGT-3’) &  TH2928 (5’-AATACTGCTCCTATAGATAG-3’). | 55.7 |
| cyt2^a,c^ | TL2-J-3037 (5’-TGGCAGATTAGTGCAATGG-3’) &  TK-N-3785 (5’-GTTTAAGAGACCAGTACTTG-3’). | 57.0 |

**a** designed by Liu & Beckenbach [14]; **b** designed by Aanen *et al.* [15]; **c** designed by Simon *et al.* [16].

Note: Primer set B was developed in our laboratory to amplify the 16S gene in samples that failed to be amplified with primer set A. The cyt1 gene was amplified as two fragments using either primer set C or D.
