# Supplementary material for: Evidence for a Higher Number of Species of Odontotermes (Isoptera) than Currently Known from Peninsular Malaysia from Mitochondrial DNA Phylogenies
Source: PLoS One. 2011 Jun 8;6(6):e20992. doi: 10.1371/journal.pone.0020992 (PMC3110805; doi:10.1371/journal.pone.0020992)
Supplement: Table S2 — List of specimens used in the analysis and their GenBank accession numbers. (DOCX) [file pone.0020992.s004.docx]

**Table S2.** List of specimens used in the analysis and their GenBank accession numbers.

|  |  |  | **GenBank accession number / Haplotype assignment (H)*** | | | |
| --- | --- | --- | --- | --- | --- | --- |
| **Species name** | **Collection site** | **Voucher number** | **16S rRNA** | **H** | **COI** | **H** |
| *O. sarawakensis* | Mt Angsi, P.M. | AG10 | GU254097 | 1 | GU254068 | i |
| *O. sarawakensis* | Mt Angsi, P.M. | AG20 | GU254119 | 1 | GU254143 | i |
| *O. sarawakensis* | Mt Korbu, P.M. | KB19 | GU254126 | 1 | GU254080 | ii |
| *O. sarawakensis* | Kledang Saiong, P.M. | KLE11 | GU254127 | 1 | GU254149 | i |
| *O. escherichi* | Mt Liang, P.M. | GL5 | GU254099 | 2 | GU254070 | iii |
| *O. hainanensis* | Taiping Lake Gdn, P.M. | S03 | GU254116 | 3 | GU254092 | iv |
| *O. hainanensis* | China |  | EU253760 |  |  |  |
| *O*. sp. 1 | Mt Korbu, P.M. | KB9 | GU254107 | 4 | GU254079 | v |
| *O*. sp. 1 | Mt Korbu, P.M. | KB22 | GU254108 | 5 | GU254081 | vi |
| *O*. sp. 1 | Maxwell Hill, P.M. | S030 | GU254118 | 6 | GU254094 | vii |
| *O.* sp. 3 | Mt Korbu, P.M. | KB5 | GU254105 | 7 | GU254078 | viii |
| *O.* sp. 3 | Mt Korbu, P.M. | KB6 | GU254106 | 8 | GU254146 | viii |
| *O.* sp. 3 | Kledang Saiong, P.M. | KLE23 | GU254129 | 8 | GU254150 | viii |
| *O.* sp. 3 | Kledang Saiong, P.M. | KLE27 | GU254131 | 8 | GU254152 | viii |
| *O.* sp. 3 | Kledang Saiong, P.M. | KLE28 | GU254132 | 8 | GU254153 | viii |
| *O.* sp. 3 | Kledang Saiong, P.M. | KLE1 | GU254109 | 9 | GU254082 | ix |
| *O.* sp. 3 | Maxwell Hill, P.M. | MX2 | GU254138 | 9 | GU254158 | ix |
| *O.* sp. 3 | Maxwell Hill, P.M. | MX5 | GU254139 | 9 | GU254159 | ix |
| *O.* sp. 3 | Mt Angsi, P.M. | AG1 | GU254096 | 10 | GU254067 | x |
| *O.* sp. 3 | Mt Liang, P.M. | GL45 | GU254103 | 11 | GU254076 | xi |
| *O.* sp. 3 | Mt Liang, P.M. | GL47 | GU254123 | 11 | GU254145 | xi |
| *O. malaccensis* | Kledang Saiong, P.M. | KLE15 | GU254128 | 12 | GU254084 | xii |
| *O. malaccensis* | Kledang Saiong, P.M. | KLE30 | GU254134 | 12 | GU254155 | xii |
| *O. malaccensis* | Kledang Saiong, P.M. | KLE32 | GU254113 | 13 | GU254156 | xii |
| *O. malaccensis* | Kledang Saiong, P.M. | KLE37 | GU254137 | 12 | GU254157 | xii |
| *O. malaccensis* | Maxwell Hill, P.M. | MX3 | GU254114 | 14 | GU254089 | xiii |
| *O. malaccensis* | Maxwell Hill, P.M. | S023 | GU254141 | 14 | GU254160 | xiii |
| *O.* sp. 2 | Penang , P.M. | SO1 | GU254115 | 15 | GU254091 | xiv |
| *O.* sp. 2 | Penang , P.M. | S050 | GU254142 | 15 | GU254161 | xiv |
| *O.* sp. 2 | Taiping Lake Gdn, P.M. | S010 | GU254117 | 16 | GU254093 | xv |
| *O.* sp. 2 | Sg. Petani, P.M. | S072 | GU254095 | 17 | GU254162 | xvi |
| *O. oblongatus* | Mt Liang, P.M. | GL17 | GU254121 | 18 | GU254075 | xvii |
| *O. paraoblongatus* | Mt Liang, P.M. | GL10 | GU254100 | 19 | GU254071 | xviii |
| *O. javanicus* | Kledang Saiong, P.M. | KLE21 | GU254112 | 20 | GU254085 | xix |
| *O. javanicus* | Kledang Saiong, P.M. | KLE24 | GU254130 | 20 | GU254151 | xix |
| *O. javanicus* | Kledang Saiong, P.M. | KLE29 | GU254133 | 20 | GU254154 | xix |
| *O. longignathus* | Mt Liang, P.M. | GL13 | GU254102 | 21 | GU254073 | xx |
| *O. longignathus* | Mt Liang, P.M. | GL20 | GU254122 | 21 | GU254144 | xx |
| *O. longignathus* | Mt Korbu, P.M. | KB1 | GU254104 | 22 | GU254077 | xxi |
| *O. longignathus* | Mt Korbu, P.M. | KB14 | GU254124 | 22 | GU254147 | xxii |
| *O. longignathus* | Mt Korbu, P.M. | KB15 | GU254125 | 22 | GU254148 | xxii |
| *O. formosanus* | China | - | AY493440 |  |  |  |
| *Mi. pakistanicus* | Penang, P.M. | - | AY302718 |  |  |  |
| *Mi. obesi* | Africa | - | EU306616 |  |  |  |
| *Ma. falciger* | Kenya | - | AF303173 |  |  |  |
| *Amitermes* sp. | Pelusi, Malawi | - | EU237168 |  |  |  |
| *Amitermes* sp. | Baringo, Kenya | - | EU237154 |  |  |  |
| *Microcerotermes* sp. | Kenya: Baringo | - | EU237182 |  |  |  |
| *Cubitermes* sp. | Thuchilla, Malawi | - | EU237156 |  |  |  |
| *O. oblongatus* | Kalimantan, Indonesia | dka162 | - |  | AY127739 |  |
| *O. hainanensis* | China |  | - |  | EU253857 |  |
| *O. minutus* | Kalimantan, Indonesia | dka161 | - |  | AY127738 |  |
| *O. javanicus* | Kalimantan, Indonesia | dka165 | - |  | AY127741 |  |
| *Ma. subhyalinus* | Kenya | BYU IGC IS95 | EU253759 |  | EU253856 |  |
| *Ma. malaccensis* | Kalimantan, Indonesia | dka160 | - |  | AY127748 |  |
| *Mi.* sp. za9 |  | - | - |  | AY818069 |  |
| *Mi.* sp. za31 |  | - | - |  | AY818074 |  |
| *Mi.* sp. za49 |  | - | - |  | AY818075 |  |
| *Mi. obesi* |  | - | - |  | AY818080 |  |
| *Amitermes evuncifer* | Niokoulo Koba, Senegal | dka97 | - |  | AY127718 |  |
| *Cubitermes* sp. | Mbalmayo, Cameroon | dka101 | - |  | AY127719 |  |
| *O. sarawakensis* | Kalimantan, Indonesia | dka167 | - |  | AY127743 |  |
| *O. billitoni* | Kalimantan, Indonesia | dka168 | - |  | AY127744 |  |

*Identical or haplotype sequences of the 16S were all given the same arabic numerals while identical sequences of the cyt1 were given the same roman numerals (for the Peninsular Malaysian samples only).

Note: Peninsular Malaysia was abbreviated as P.M.; *Macrotermes* as *Ma*.; and *Microtermes* as *Mi.*
