# Supplementary material for: Evidence for a Higher Number of Species of Odontotermes (Isoptera) than Currently Known from Peninsular Malaysia from Mitochondrial DNA Phylogenies
Source: PLoS One. 2011 Jun 8;6(6):e20992. doi: 10.1371/journal.pone.0020992 (PMC3110805; doi:10.1371/journal.pone.0020992)
Supplement: Table S3 — Nucleotide differences between 16S sequences of Odontotermes species. (DOCX) [file pone.0020992.s005.docx]

**Table S3.** Nucleotide differences between 16S sequences of *Odontotermes* species*.*

| No. | Species | 1 | 2 | 3 | 4 | 5 | 6 | 7 | 8 | 9 | 10 | 11 |
| --- | --- | --- | --- | --- | --- | --- | --- | --- | --- | --- | --- | --- |
| 1. | *O. sarawakensis* | - | 5.74 | 5.75 | 4.91 | 4.29 | 3.48 | 3.71 | 4.63 | 4.31 | 4.59 | 3.75 |
| 2. | *O. escherichi* | 28 | - | 6.57 | 5.32 | 5.12 | 5.12 | 4.94 | 5.68 | 3.68 | 5.22 | 3.75 |
| 3. | *O. paraoblongatus* | 28 | 32 | - | 5.52 | 4.92 | 4.94 | 5.58 | 6.13 | 5.13 | 6.08 | 4.36 |
| 4. | *O. longignathus* | 24 | 26 | 29 | - | 4.29 | 4.30 | 4.12 | 4.84 | 4.10 | 5.43 | 3.95 |
| 5. | *O. oblongatus* | 21 | 25 | 24 | 21 | - | 2.66 | 2.68 | 3.99 | 4.93 | 4.34 | 3.12 |
| 6. | *O*. sp. 1 | 17 | 25 | 24 | 21 | 13 | - | 1.44 | 3.78 | 4.31 | 4.17 | 2.92 |
| 7. | *O. malaccensis* | 18 | 24 | 27 | 20 | 13 | 7 | - | 3.38 | 4.12 | 3.98 | 3.15 |
| 8. | *O. javanicus* | 22 | 27 | 29 | 23 | 19 | 18 | 16 | - | 4.85 | 0.63 | 3.58 |
| 9. | *O.* sp. 3 | 21 | 18 | 25 | 20 | 24 | 21 | 20 | 23 | - | 4.81 | 3.13 |
| 10. | *O.* sp. 2 | 22 | 25 | 29 | 26 | 21 | 20 | 19 | 3 | 23 | - | 3.55 |
| 11. | *O. hainanensis* | 18 | 18 | 21 | 19 | 15 | 14 | 15 | 17 | 15 | 17 | - |

Note: The figures below the diagonal are absolute number of base pair differences while the numbers above the diagonal are percentage differences among pairs of species.
