# Supplementary material for: Evidence for a Higher Number of Species of Odontotermes (Isoptera) than Currently Known from Peninsular Malaysia from Mitochondrial DNA Phylogenies
Source: PLoS One. 2011 Jun 8;6(6):e20992. doi: 10.1371/journal.pone.0020992 (PMC3110805; doi:10.1371/journal.pone.0020992)
Supplement: Table S4 — Nucleotide differences between cyt1 sequences of Odontotermes species. (DOCX) [file pone.0020992.s006.docx]

**Table S4.** Nucleotide differences between cyt1 sequences of *Odontotermes* species.

| No. | Species | 1 | 2 | 3 | 4 | 5 | 6 | 7 | 8 | 9 | 10 | 11 |
| --- | --- | --- | --- | --- | --- | --- | --- | --- | --- | --- | --- | --- |
| 1. | *O. sarawakensis* | - | 5.32 | 7.29 | 6.91 | 7.14 | 6.01 | 6.38 | 6.10 | 6.25 | 6.22 | 6.05 |
| 2. | *O. escherichi* | 51 | - | 6.67 | 5.77 | 5.31 | 5.43 | 5.20 | 5.89 | 5.30 | 5.91 | 5.42 |
| 3. | *O. paraoblongatus* | 70 | 64 | - | 6.96 | 6.59 | 7.86 | 7.11 | 6.62 | 6.46 | 6.74 | 6.37 |
| 4. | *O. longignathus* | 66 | 55 | 67 | - | 7.08 | 7.44 | 6.32 | 5.73 | 6.49 | 5.75 | 6.75 |
| 5. | *O. oblongatus* | 66 | 49 | 62 | 66 | - | 5.31 | 6.14 | 6.24 | 6.71 | 6.35 | 5.32 |
| 6. | *O*. sp. 1 | 52 | 47 | 68 | 64 | 44 | - | 6.39 | 6.79 | 6.94 | 6.93 | 6.49 |
| 7. | *O. malaccensis* | 59 | 48 | 67 | 59 | 58 | 53 | - | 6.54 | 6.92 | 6.65 | 5.42 |
| 8. | *O. javanicus* | 57 | 55 | 63 | 54 | 59 | 57 | 62 | - | 6.10 | 0.10 | 5.47 |
| 9. | *O.* sp. 3 | 60 | 51 | 62 | 62 | 62 | 60 | 64 | 57 | - | 6.22 | 6.65 |
| 10. | *O.* sp. 2 | 58 | 55 | 64 | 54 | 60 | 58 | 63 | 1 | 58 | - | 5.59 |
| 11. | *O. hainanensis* | 58 | 52 | 62 | 65 | 50 | 56 | 51 | 52 | 64 | 53 | - |

Note: The figures below the diagonal are absolute number of base pair differences while the numbers above the diagonal are percentage differences among pairs of species.
